# Comparative phylogenetic analysis of complete plastid genomes of *Renanthera* (Orchidaceae)

**DOI:** 10.3389/fgene.2022.998575

**Published:** 2022-09-14

**Authors:** Tao Xiao, Liefen He, Liangliang Yue, Yonghong Zhang, Shiou Yih Lee

**Affiliations:** ^1^ School of Life Sciences, Yunnan Normal University, Kunming, China; ^2^ Yunnan Key Laboratory of Plateau Wetland Conservation, Restoration and Ecological Services, Southwest Forestry University, Kunming, China; ^3^ Faculty of Health and Life Sciences, INTI International University, Nilai, Malaysia

**Keywords:** renanthera, chloroplast genome, comparative analysis, phylogenomics, vandaeae

## Abstract

Owing to its attractive flower shape and color, *Renanthera* (Orchidaceae), comprising about 19 species, has significant ornamental value as a houseplant, in floral design and in landscape gardens. Two species of *Renanthera* are categorized as endangered and critically endangered in China’s Red List and international trade in these orchids is currently strictly monitored by the Convention on International Trade in Endangered Species of Wild Fauna and Flora (CITES). This paper reports on the *de novo* assembled and annotated plastome of four species of *Renanthera*; *R. citrina*, *R. coccinea*, *R. imschootiana*, and *R. philippinensis*. The length of the plastome sequences ranged from 144,673 bp (*R. imschootiana*) to 149,007 bp (*R. coccinea*) with GC content of 36.6–36.7%. The plastomes showed a typical quadripartite structure, including a large single-copy (84,241–86,404 bp), a small single-copy (11,468–12,167 bp), and a pair of inverted repeats (24,482–25,715 bp) regions. Of the 120 genes detected, 74 were protein coding, 38 were tRNA, and eight were rRNA genes. The plastome of *Renanthera* is rather conserved, but nucleotide variations that could distinguish them apart are noticeable—the total number of tandem repeats ranged from 62 (in *R. imschootiana*) to 74 (in *R. citrina*); while the number of long repeats ranged from 21 (in *R. imschootiana* and *R. philippinensis*) to 43 (in *R. citrina*). Three hypervariable regions (*psb*I*-trn*S-GCU, *trn*G-GCC, *rpl*32) were identified. Phylogenetic analyses based on the CDS using maximum likelihood (ML) and Bayesian inference (BI) revealed that *Renanthera* is closely related to *Holcoglossum*, *Neofinetia*, *Pendulorchis*, and *Vanda*. The relationship between the four species of *Renanthera* was fully resolved; a monophyletic clade was formed and *R. coccinea* was recorded as the first to diverge from the rest. The genetic data obtained from this study could serve as a useful resource for species identification in *Renanthera* as well as contribute to future research on the phylogenomics of Orchidaceae.

## Introduction

*Renanthera* Lour. (Aeridinae, Vandeae, Orchidaceae) comprises about 19 species of epiphytic and/or lithophytic perennial herbs, mainly distributed in China, India, Indonesia, Malaysia, New Guinea, Philippines Vietnam, and the Solomon Islands (Chen and Jeffrey, 2009; Chase et al., 2015). Members of *Renanthera* produce a spectacular branched inflorescence containing numerous flowers with large, yellow, orange and red lateral sepals (Luo et al., 2012). Due to the magnificent floral features, these orchids have received great attention by orchid breeders and collectors as houseplants, floral design, and landscape gardening. However, their over-exploitation in the wild has disturbed their original habitat severely and threatened their survival in the wild. As a result, all members of *Renanthera*, except for *R. imschootiana*, which is listed in of the Convention on International Trade in Endangered Species of Wild Fauna and Flora (CITES) since 1975 (UNEP-WCMC, 2020). In China, three species are recorded, including *R. citrina*, *R. coccinea*, and *R. imschootiana* (Chen and Jeffrey, 2009); however, two of them, *R. coccinea*, and *R. imschootiana* are categorized as endangered and critically endangered, respectively, in the Red List of China Higher Plants (Qin et al., 2017).

In general, the morphological features, such as the number of pollinia, presence or absence of a nectar spur, shape and size of labellum, and shape of the column-foot, have been used as key characters in the taxonomic classification of Aeridinae (Pridgeon et al., 2014). However, the true phylogenetic relationships at subtribe level were unclear due to multiple evolutionary stages in the evolution of character states over a short period of time (Topik et al., 2005). Genetic studies on *Renanthera* are limited. Previous molecular phylogenetic studies of Aeridinae using a combined dataset of four plastid regions (*atp*I-*atp*H, *mat*K, *psb*A-*trn*H, *trn*L-*trn*F) and the nuclear ribosomal internal transcribed spacer (ITS) region reportedly resolved the phylogenetic relationship within *Renanthera* (Zou et al., 2015). However, the phylogenetic tree was not supported with a strong backbone. The lack of informative sites resulted in a number of uncertainties in the molecular placements of many genera in the subtribe.

With the development of next-generation sequencing (NGS) technology, sequencing of the complete organellar genome has become simpler and affordable. The use of genome-scale data to resolve relationships in complicated plant groups appears promising. Coupled with advanced bioinformatic analytic tools, the phylogenetic relationships of many members of Orchidaceae, which has been uncertain for many years, were resolved (Li et al., 2019; Kim et al., 2020a). The complete plastid genome (plastome) sequences of 10 taxa of Vandeae, Kim et al. (2020b) revealed the molecular placement of Vandeae in the Epidendroideae. The phylogenetic relationship among members in Vandeae, with a focus on Aeridinae, was further identified in a phylogenetic reconstruction based on complete plastome sequences (Liu et al., 2020). Despite the well-supported tree reported by Liu et al. (2020), members of *Renanthera* were not included.

As an understudied yet threatened species in China, three species that are native to China, including *R. citrina*, *R. coccinea*, and *R. imschootiana*, as well as a domesticated species, *R. philippinensis*, were included in this study. To provide genetic data at genome level, we sequenced and characterized the complete plastomes of four species of *Renanthera*. At the same time, we analyzed the sequence repeats, the contraction and expansion of the inverted repeat (IR) region, identified the variable sites, and revealed the phylogenetic relationships between the four species of *Renanthera* to further elucidate evolutionary patterns in *Renanthera* at the plastome level. The findings obtained through this study yielded useful genomic information on these threatened species that may aid in conservation strategies.

## Materials and methods

### Plant material, sequencing, and genome assembly

Four *Renanthera* species, including *R. citrina*, *R. coccinea*, *R. imschootiana*, and *R. philippinensis*, were collected from natural populations, except for *R. philippinensis* which is a cultivated species from an orchid nursery in Guangdong. The orchids are transferred and planted in the greenhouse of Yunnan Normal University, Kunming, China (24°52'1.41“N, 102°51'19.39”E). Voucher specimens are deposited in the Herbarium of Yunnan Normal University (YNUB) (Table 1). Total genomic DNA was extracted from fresh leaves using the E.Z.N.A® HP Plant DNA Kit (Omega Bio-tek, GA, United States) based on the manufacturer’s protocol. Shotgun libraries (350 bp) were prepared using a TruSeq DNA Sample Prep Kit (Illumina, United States). Next-generation sequencing (NGS) was conducted on an Illumina Hiseq 2000 sequencing platform (Illumina, CA, United States), from which approximately 6 Gb of raw NGS data was obtained. The raw reads were filtered using the NGS QC Toolkit (Patel and Jain, 2012) to remove low quality reads and to trim off the sequence adaptors. The filtered reads were then fed into the GetOrganelle (Jin et al., 2020) pipeline for genome *de novo* assembly. Gene annotation was carried out using GeSeq (Tillich et al., 2017) based on default parameters, and was checked through Geneious Prime v2020.0.3 (Kearse et al., 2012). The circular map of the genome was illustrated using OGDRAW (Greiner et al., 2019).

**TABLE 1:** Complete plastid genome features of four species of *Renanthera*.

Features	*R. citrina*	*R. coccinea*	*R. imschootiana*	*R. philippinensis*
Locality	Mount Wutong, Guangdong	Malipo County, Yunnan	Yuanjiang County, Yunnan	Guangdong Xiuli Orchid Co., Ltd, Guangdong
Coordinate	22˚34’, 114˚12’	23˚7’, 104˚42’	23˚35’, 101˚59’	23˚6’, 113˚12’
Collector name	Yonghong Zhang	Yonghong Zhang	Yonghong Zhang	Yonghong Zhang
Voucher number	OR-001	OR-002	OR-003	OR-004
Size (base pair; bp)	146,255	149,007	144,673	147,713
GC content (%)	36.6	36.7	36.7	36.7
LSC length (bp)	84,821	85,546	84,241	86,404
GC content (%)	33.9	34.0	33.9	34.0
SSC length (bp)	11,896	12,031	11,468	12,167
GC content (%)	28.0	28.2	27.9	27.7
IR length (bp)	24,769	25,715	24,482	24,571
GC content (%)	43.3	43.3	43.5	43.5
Number of genes	120	120	120	120
Protein-coding genes	74	74	74	74
tRNA genes	38	38	38	38
rRNA genes	8	8	8	8
Accession number	OK377033	OK377034	OK377035	OK377036

### Identification of sequence repeats

Simple sequence repeats (SSRs) were calculated using MISA-web (Beier et al., 2017), in which the parameters for SSR identification were set at minimal repeat numbers of 10, 5, 4, 3, 3, and 3 for mono-, di-, tri-, tetra-, penta- and hexa- nucleotide repeats, respectively. For large repeats, REPuter was used to identify forward, palindrome, reverse, and complementary long repeats (Kurtz and Schleiermacher, 1999). The hamming distance was set as 3, while the minimal repeat size was 30 bp.

### Codon usage analysis

The nucleotide sequence of each protein-coding gene in the plastome was extracted using Geneious Prime v2020.0.3 (Kearse et al., 2012). The relative synonymous codon usage (RSCU) for each protein-coding gene was calculated using MEGA7 (Kumar et al., 2016).

### Comparative genome analysis, IR border and divergence analyses

Chloroplast genomes across the four species of *Renanthera* were compared on the mVISTA tool (Frazer et al., 2004) using Shuffle-LAGAN mode with the sequence of *Renanthera coccinea* as a reference. To detect the expansion and contraction of the inverted repeat (IR) region, the boundaries between the IR and single-copy regions (IR/SC) of the plastomes were visualized using IRscope (Amiryousefi et al., 2018). Prior to nucleotide divergence analysis, the plastome sequences were aligned using MAFFT v7 (Katoh and Standley, 2013). The nucleotide variability of the four plastomes of *Renanthera* were calculated using DnaSP v5.10 (Librado and Rozas, 2009), in which highly mutational hotspot regions were identified through a sliding window analysis. The length of the window frame was set at 1,000 bp and a 500 bp step size was selected. The number and location of the single nucleotide polymorphisms (SNPs) and variable sites in the plastome alignment were also determined using the same program.

### Phylogenetic reconstruction

Phylogenetic analysis was carried out based on CDSs of 30 species of Orchidaceae. The CDSs were extracted, ClustalW-aligned, and concatenated alphabetically using Geneious Prime v2020.0.3 (Kearse et al., 2012). Three closely related species, *Tridactyle tridactylites* (Angraecinae, Vandeae; MW760855), *Cattleya crispata* (Epidendroideae; KP168671), and *Cymbidium ensifolium* (Epidendroideae; KT722983) were included as outgroups. Phylogenetic trees were constructed using two methods: maximum likelihood (ML) and Bayesian inference (BI). For ML, the concatenated dataset was fed into the RAxML v8.2.11 pipeline (Stamatakis, 2014) and analysis was carried out based on general time-reversible (GTR) with the gamma distributed (+G) (=GTR + G) substitution model. Each branch node was assessed under 1,000 bootstrap replicates. For BI, the concatenated dataset was fed into the MrBayes v3.2.6 pipeline (Ronquist et al., 2012), in which a mix substitution type and a 4 by 4 nucleotide substitution model was selected for the likelihood model. Markov chain Monte Carlo simulation was performed twice independently for 2,000,000 generations. Four chains were selected and data sampling was conducted every 100 generations. The first 25% of trees was discarded as burn-in. Both pipelines are available in the CIPRES Science Gateway (Miller et al., 2011). Final tree results for both analyses were visualized using FigTree v1.4.4 (Rambaut, 2018).

## Results

### Genome features

**FIGURE 1:**
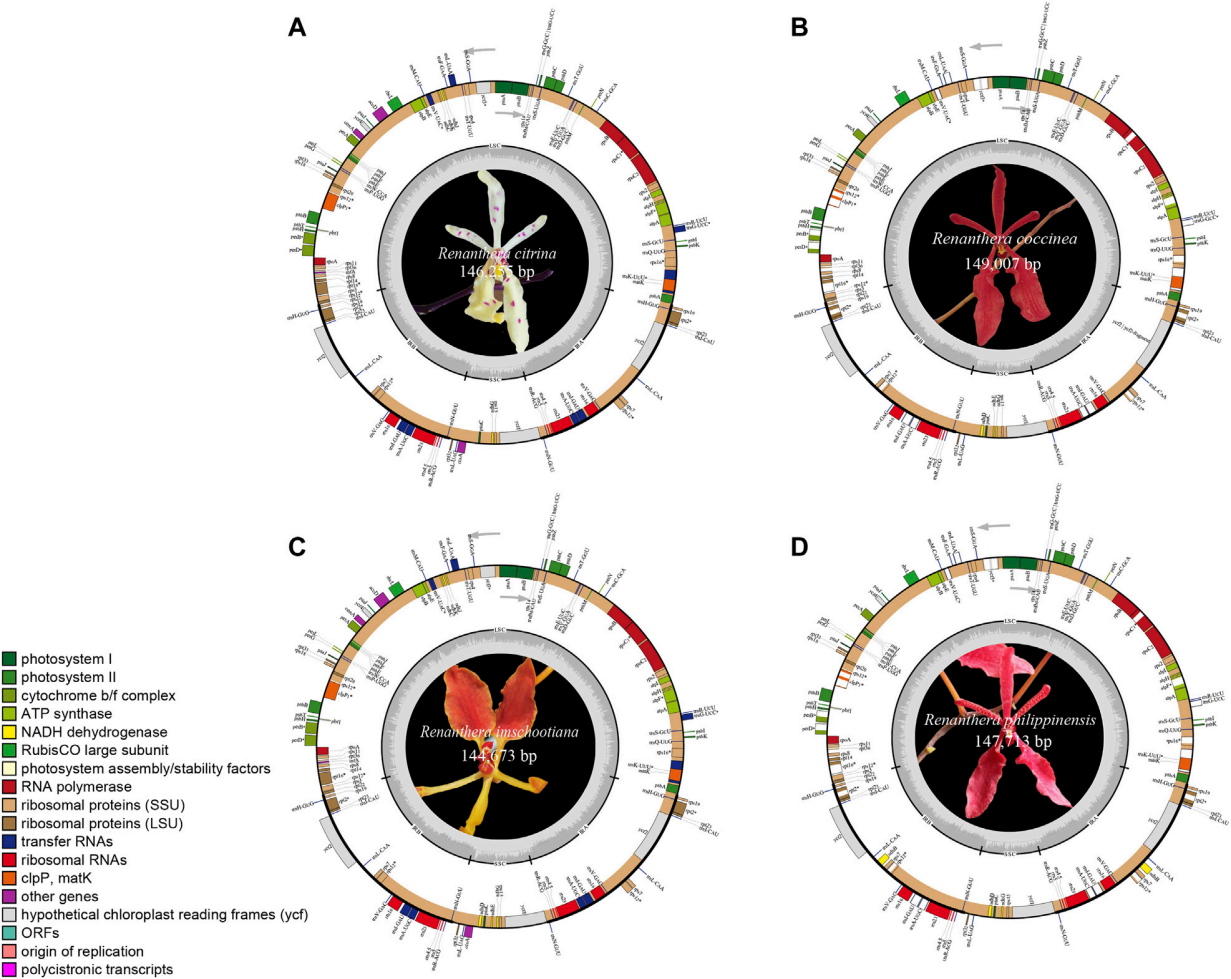
Gene map of plastid genomes of four species of *Renanthera* used in this study, including **(A)** *R. citrina*; **(B)** *R. coccinea*; **(C)** *R. imschootiana*; **(D)** *R. philippinensis*. Genes on inside of map are transcribed in clockwise direction; genes on outside of map are transcribed in counterclockwise direction. Different functional groups of genes are shown in different colors. Inverted repeat (IR), small single-copy (SSC), and large single-copy (LSC) regions are indicated.

The plastomes of the four species of *Renanthera* displayed a typical quadripartite structure that consisted of a genome size ranging from 144,673 bp (*R. imschootiana*) to 149,007 bp (*R. coccinea*) (Figure 1). The plastomes contained a pair of IRs that were separated by a large single-copy (LSC) region and a small single-copy (SSC) region. The size of each region varied across all species (Table 1). For the IR region, the size ranged from 24,482 bp (*R. imschootiana*) to 25,715 bp (*R. coccinea*); while the SSC and the LSC regions were 11,468 bp (*R. imschootiana*) to 12,167 bp (*R. philippinensis*) and 84,241 bp (*R. imschootiana*) to 86,404 bp (*R. philippinensis*), respectively. The overall GC contents for all the plastomes were 36.7%, except for *R. citrina*, which was 36.6%. All four plastomes were predicted with the same number of genes, which was 120. The genes include 74 CDS, 38 rRNA, and eight tRNA genes. Among these genes, 17 were duplicates in the IR regions, including five CDS (*rpl*2, *rpl*23, *rps*7, *rps*19, *ycf*2), eight tRNA (*trn*A-UGC, *trn*H-GUG, *trn*I-CAU, *trn*I-GAU, *trn*L-CAA, *trn*N-GUU, *trn*R-ACG, *trn*V-GAC), and four rRNA (*rrn*4.5, *rrn*5, *rrn*16, *rrn*23) genes (Table 2).

**TABLE 2:** Genes present in the plastid genomes of four species of *Renanthera* used in this study.

	Genes
RNAs, ribosomal	*rrn*4.5 (×2), *rrn*5 (×2), *rrn*16 (×2)*, rrn*23 (×2)
RNAs, transfer	*trn*A-UGC (×2)*, *trn*C-GCA, *trn*D-GUC, *trn*E-UUC, *trn*F-GAA, *trn*fM-CAU, *trn*G-GCC*, *trn*G-UCC, *trn*H-GUG (×2), *trn*I-CAU(×2), *trn*I-GAU (×2)*, *trn*K-UUU *, *trn*L-CAA (×2), *trn*L-UAA*, *trn*L-UAG, *trn*M-CAU, *trn*N-GUU(×2), *trn*P-UGG, *trn*Q-UUG, *trn*R-ACG (×2), *trn*R-UCU, *trn*S-GCU, *trn*S-GGA, *trn*S-UGA, *trn*T-GGU, *trn*T-UGU, *trn*V-GAC (×2), *trn*V-UAC*, *trn*W-CCA, *trn*Y-GUA
Transcription and splicing	*mat*K, *rpo*A, *rpo*B, *rpo*C1*, *rpo*C2
Translation, ribosomal proteins	
Small subunit	*rps2*, *rps*3, *rps*4, *rps*7 (×2), *rps*8, *rps*11, *rps*12*, *rps*14, *rps*15, *rps*16*, *rps*18, *rps*19 (×2)
Large subunit	*rpl*2 (×2) *, *rpl*14, *rpl*16*, *rpl*20, *rpl*22, *rpl*23 (×2), *rpl*32, *rpl*33, *rpl*36
Photosynthesis	
ATP synthase	*atp*A, *atp*B, *atp*E, *atp*F*, *atp*H, *atp*I
Photosystem I	*psa*A, *psa*B, *psa*C, *psa*I, *psa*J
Photosystem II	*psb*A, *psb*B, *psb*C, *psb*D, *psb*E, *psb*F, *psb*H, *psb*I, *psb*J, *psb*K, *psb*L, *psb*M, *psb*N, *psb*T, *psb*Z
Calvin cycle	*rbc*L
Cytochrome complex	*pet*A, *pet*B*, *pet*D*, petG, *pet*L, *pet*N
Others	*acc*D, *ccs*A, *cem*A, *clp*P**, infA*, ycf*1, *ycf*2 (×2), *ycf*3**, *ycf*4

*Genes with one intron.

**Genes with two introns.

×2 duplicated genes.

### Repeats analyses

**FIGURE 2:**
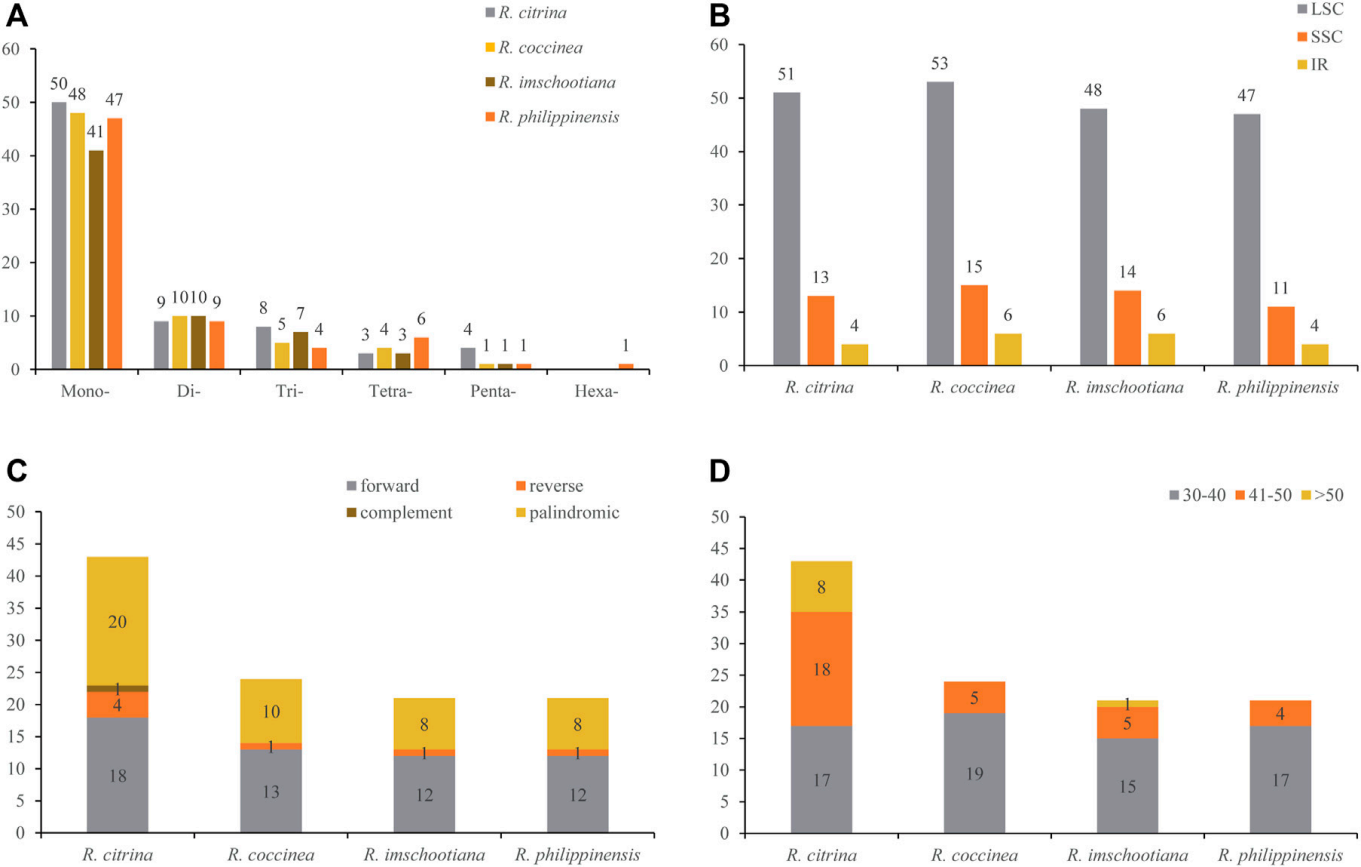
Distribution of sequence repeats in the plastid genomes of four species of *Renanthera*. **(A)** Number of different short sequence repeat (SSR) types detected in four plastid genomes of *Renanthera*; **(B)** Frequencies of identified SSRs in large single-copy (LSC), small single-copy (SSC) and inverted repeat (IR) regions; **(C)** Number of long repeats based on types; **(D)** Number of long repeats by sequence length.

SSRs analysis detected 74, 68, 62, and 68 SSRs in *R. citrina*, *R. coccinea*, *R. imschootiana* and *R. philippinensis*, respectively (Figure 2). Among the SSRs, the mononucleotide repeats were the most abundant; at least 41–50 mononucleotide repeats were in the four species. This was followed by the dinucleotides (9–10 repeats), trinucleotides (4—8 repeats), and tetranucleotides (3—6 repeats). For pentanucleotide repeats, all the species was recorded with one occurrence, except *R. citrina*, which had four. One hexanucleotide repeat was detected in *R. philippinensis*, but none in the others. Most of the SSRs were located in the LSC region, in which 51 (75%), 53 (71.62%), 48 (70.59%), and 47 (75.81%) were recorded in *R. citrina*, *R. coccinea*, *R. imschootiana* and *R. philippinensis*, respectively.

The number of long repeats detected in the four plastomes ranged from 21 (*R. imschootiana* and *R. philippinensis*) to 43 (*R. citrina*). All the plastomes contained three types of long repeats that were forward, reverse, and palindromic, except for *R. citrina*. For *R. citrina*, aside from the three long repeat types, it also contained a complement type. All species, except for *R. citrina*, had long repeats mostly in the 30–40 bp range; the long repeat of *R. citrina* was 41–50 bp. For long repeats above 50 bp, *R. citrina* and *R. imschootiana* had eight and one, respectively; none was in *R. coccinea* and *R. philippinensis*.

### Codon usage analysis

A total of 184,687 codons was identified in the CDSs predicted across the four plastomes of *Renanthera* (Table 3). Among these codons, leucine (10.7%) and isoleucine (9.1%) were the most abundant amino acids, whereas cysteine (2.4%) had the lowest frequency. All four plastomes exhibited similar codon preferences, in which 32 codons were more preferred (RSCU >1), and 30 codons were least preferred (RSCU <1). No preference (RSCU = 1) was found for tryptophan (UGG) or methionine (AUG). For start codons, methionine (AUG) was more preferred than tryptophan (UGG); while for stop codons, UAA was more preferred than UAG and UGA, in the four different plastomes.

**TABLE 3:** Relative synonymous codon usage values (RSCU) in plastid genome of four species of *Renanthera*.

Amino acid	Codon	*R. citrina*		*R. coccinea*		*R. imschootiana*		*R. philippinensis*	
		Count	RSCU	Count	RSCU	Count	RSCU	Count	RSCU
Alanine	GCU	455	1.31	391	1.17	452	1.3	441	1.31
	GCC	321	0.92	339	1.01	302	0.87	303	0.9
	GCA	417	1.2	412	1.23	428	1.23	409	1.21
	GCG	198	0.57	200	0.6	207	0.6	198	0.59
Arginine	CGU	345	0.67	328	0.63	337	0.66	357	0.69
	CGC	209	0.41	225	0.43	204	0.4	217	0.42
	CGA	506	0.99	493	0.95	483	0.95	485	0.93
	CGG	288	0.56	337	0.65	358	0.7	318	0.61
	AGA	1,162	2.26	1,125	2.17	1,111	2.18	1,156	2.22
	AGG	569	1.11	600	1.16	566	1.11	589	1.13
Asparagine	AAU	1,769	1.45	1,701	1.41	1,656	1.42	1,651	1.4
	AAC	674	0.55	708	0.59	682	0.58	702	0.6
Aspartic acid	GAU	1,037	1.43	1,036	1.44	1,066	1.46	1,058	1.45
	GAC	417	0.57	399	0.56	394	0.54	400	0.55
Cysteine	UGU	691	1.22	658	1.18	652	1.24	672	1.2
	UGC	441	0.78	462	0.83	400	0.76	446	0.8
Glutamic acid	CAA	855	1.41	923	1.37	887	1.38	954	1.38
	CAG	355	0.59	425	0.63	398	0.62	428	0.62
	GAA	1,241	1.41	1,322	1.37	1,302	1.37	1,337	1.41
	GAG	515	0.59	607	0.63	593	0.63	554	0.59
Glycine	GGU	539	1.01	529	1.04	503	1.03	493	0.97
	GGC	348	0.66	287	0.57	306	0.63	315	0.62
	GGA	775	1.46	729	1.44	703	1.44	728	1.44
	GGG	463	0.87	482	0.95	441	0.9	487	0.96
Histidine	CAU	856	1.44	914	1.39	803	1.42	857	1.4
	CAC	334	0.56	398	0.61	330	0.58	364	0.6
Isoleucine	AUU	1,766	1.23	1,631	1.16	1,694	1.21	1,647	1.2
	AUC	1,072	0.75	1,098	0.78	1,027	0.73	1,031	0.75
	AUA	1,477	1.03	1,478	1.05	1,483	1.06	1,447	1.05
Leucine	UUA	978	1.21	986	1.19	1,072	1.29	1,037	1.27
	UUG	960	1.19	1,025	1.23	1,020	1.23	974	1.19
	CUU	1,069	1.33	1,068	1.28	1,037	1.25	1,100	1.34
	CUC	617	0.77	635	0.76	579	0.7	595	0.73
	CUA	767	0.95	794	0.95	792	0.96	760	0.93
	CUG	440	0.55	482	0.58	468	0.57	445	0.54
Lysine	AAA	2,008	1.35	1,987	1.33	1,997	1.35	1,990	1.34
	AAG	962	0.65	996	0.67	968	0.65	986	0.66
Methionine	AUG	840	1	905	1	848	1	847	1
Phenylalanine	UUU	2,109	1.16	2,210	1.17	2,077	1.16	2,258	1.2
	UUC	1,533	0.84	1,567	0.83	1,506	0.84	1,514	0.8
Proline	CCU	590	1.13	598	1.14	603	1.15	623	1.16
	CCC	530	1.01	480	0.92	515	0.98	501	0.94
	CCA	673	1.28	684	1.31	653	1.24	684	1.28
	CCG	302	0.58	328	0.63	335	0.64	334	0.62
Serine	UCU	1,213	1.57	1,257	1.59	1,294	1.66	1,294	1.63
	UCC	809	1.05	859	1.08	827	1.06	817	1.03
	UCA	940	1.22	957	1.21	987	1.27	1,022	1.29
	UCG	557	0.72	539	0.68	512	0.66	482	0.61
	AGU	645	0.83	660	0.83	620	0.8	673	0.85
	AGC	472	0.61	486	0.61	426	0.55	472	0.59
Threonine	ACU	619	1.17	597	1.17	607	1.19	591	1.15
	ACC	522	0.99	535	1.05	520	1.02	548	1.06
	ACA	636	1.2	575	1.13	613	1.2	589	1.14
	ACG	339	0.64	335	0.66	303	0.59	335	0.65
Tryptophane	UGG	621	1	618	1	623	1	650	1
Tyrosine	UAU	1,452	1.4	1,466	1.35	1,386	1.41	1,425	1.36
	UAC	615	0.6	701	0.65	586	0.59	676	0.64
Valine	GUU	728	1.39	696	1.3	719	1.36	702	1.37
	GUC	383	0.73	399	0.75	382	0.72	426	0.83
	GUA	628	1.2	685	1.28	665	1.26	598	1.17
	GUG	363	0.69	359	0.67	342	0.65	324	0.63
Stop codon	UGA	982	1.08	1,044	1.06	929	1.08	1,078	1.11
	UAA	1,036	1.14	1,111	1.12	984	1.15	1,072	1.1
	UAG	718	0.79	808	0.82	661	0.77	771	0.79

### IR contraction and expansion

**FIGURE 3:**
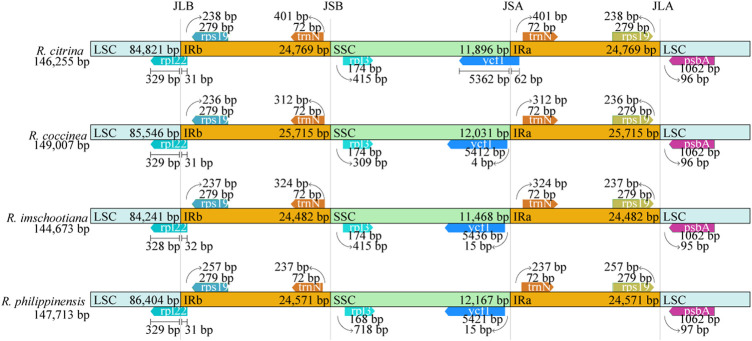
Comparison of the junctions of LSC, SSC, and IR regions among four plastid genomes of *Renanthera*.

The positions of the IR junctions were well-conserved across the four species of *Renanthera* (Figure 3). At the junction between the LSC and IRb (JLB), genes *rpl*22 and *rps*19 were adjacent to the junction; the *rpl*22 gene of LSC crossed over into IRb. For the junction between the LSC and IRa (JLA), genes *rps*19 and *psb*A were adjacent to the junction. The *rpl*32 and *trn*N genes were adjacent to the junction between SSC and IRb (JSB); while the *trn*N and *ycf*1 genes were adjacent to the junction between SSC and IRa (JSA). The *ycf*1 gene of *R. citrina* crossed over JSA, into the IRa region; while the *ycf*1 genes for the other three species were still intact within the SSC region.

### Comparative genome analysis and identification of hypervariable regions

**FIGURE 4:**
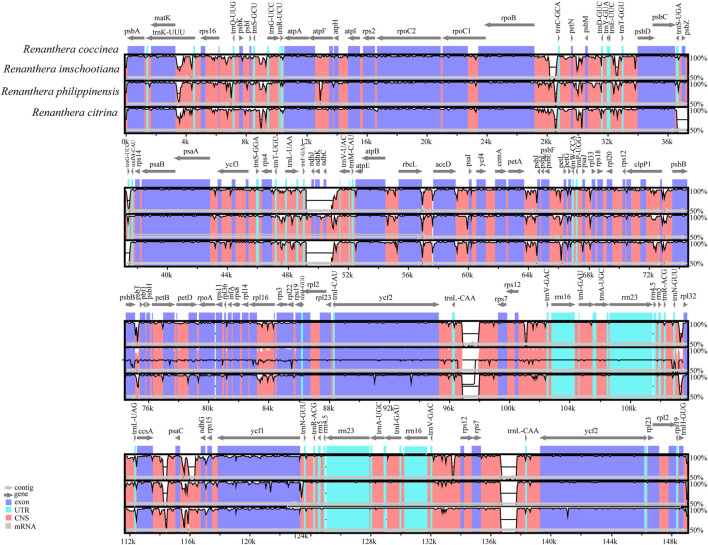
Complete plastid genome comparison of four species of *Renanthera*. The plastid genome of *R. coccinea* was selected as reference.

**FIGURE 5:**
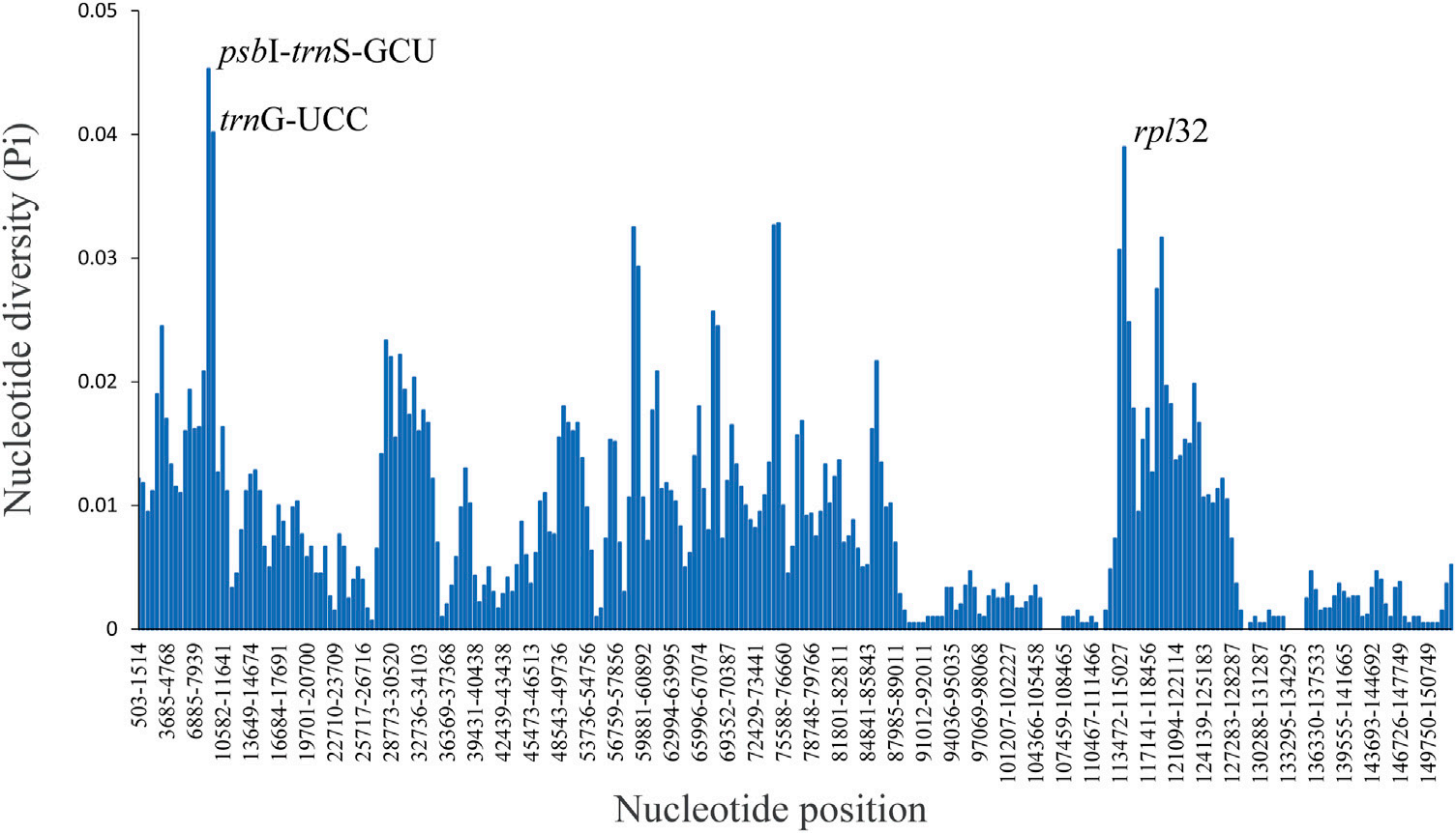
Nucleotide diversity (Pi) in the complete plastid genomes of four species of *Renanthera*. Window length: 1,000 bp, step size: 500 bp.

Comparative analysis of the complete chloroplast genome can reveal differences between different species (Figure 4). Altogether, the genome sequence alignment of the four species of *Renanthera* exhibit a high degree of similarity. Three genes, *ndh*J, *ndh*K, and *ndh*C, were obviously lost in *R. citrina* and *R. imschootiana*. Additionally, a distinct large gap was observed from the *rps*7 gene to *trn*L-CAA in the IRa and IRb of *R. citrina*, *R. imschootiana* and *R. philippinensis*. The nucleotide diversity (Pi) value for the four plastomes ranged from 0 to 0.04533, with an average of 0.0086. At a cut-off point set at Pi ≥ 0.035, three hypervariable regions, including *psb*I*-trn*S-GCU, *trn*G-GCC, *rpl*32, were identified (Figure 5). Among the three hypervariable regions, two were in the LSC region, while one was in the SSC region. None was detected in the IR region.

Plastome alignment of the four species of *Renanthera* resulted in a 152,882 bp long sequence alignment. Across the alignment, 433 parsimony informative sites and 1,838 singletons were identified. A total of 2,271 SNPs was identified. SNPs in the LSC region were the most abundant (n = 1,708), followed by 360 and 203 of them in the SSC and IR regions, respectively.

### Phylogenetic analyses

**FIGURE 6:**
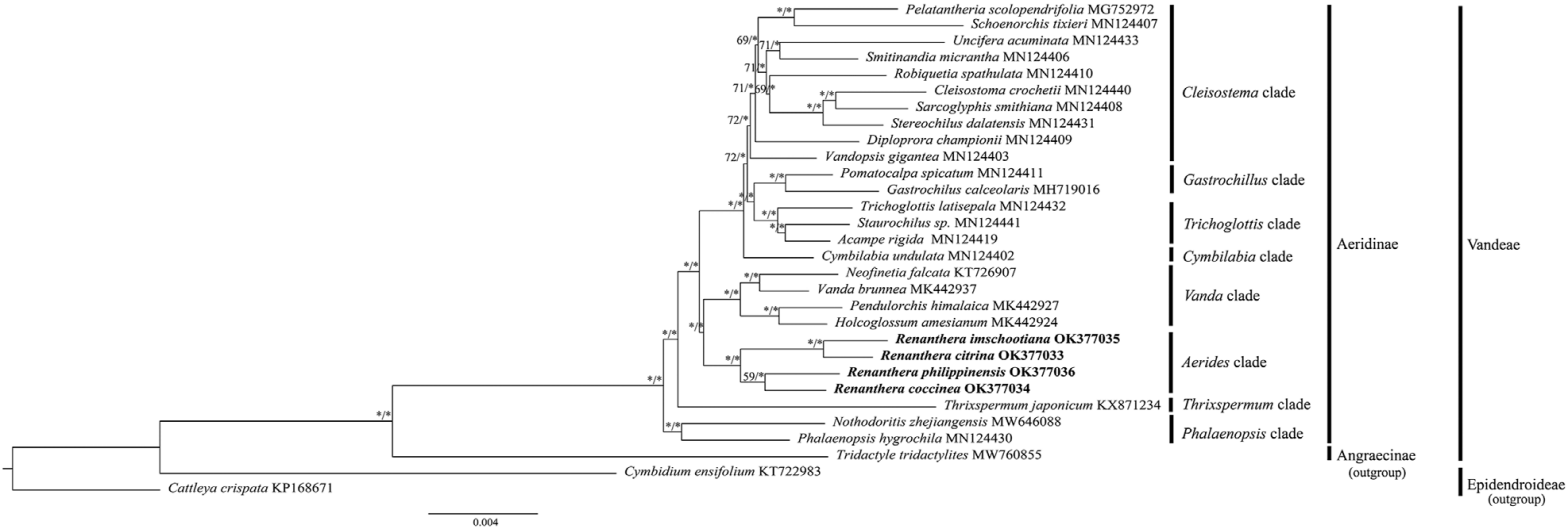
Phylogenetic trees based on CDS sequences of 30 species of Orchidaceae. Three species, *Tridactyle tridactylites* (Angraecinae, Vandeae; MW760855), *Cattleya crispata* (Epidendroideae; KP168671), and *Cymbidium ensifolium* (Epidendroideae; KT722983) were included as outgroups. Numbers on each node represent bootstrap support (BS) and posterior probability (PP) values. Branches indicated with BS ≥ 75 and PP ≥ 0.90 are considered as well-supported, which are represented with an asterisk (*).

Phylogenetic analyses using both ML and BI methods based on the CDS sequences of 30 species of Orchidaceae revealed similar topological structure in the phylogenetic trees; hence, only the ML tree is presented, with the values of the bootstrap support (BS) as well as the posterior probability (PP) of the ML and BI trees indicated on each branch node (Figure 6). In general, the phylogenetic relationship within *Renanthera* was well-resolved (BS ≥ 75%, PP ≥ 0.90); *R. coccinea* clustered with *R. philippinensis* and *R. citrina* clustered with *R. imschootiana*. *Renanthera* is closely-related to the *Holcoglossum* + *Neofinetia* + *Pendulorchis* + *Vanda* clade. In general, all the branch nodes in the phylogenetic tree were strongly supported in the BI analysis; while seven branch nodes were indicated with moderate bootstrap support values in the ML analysis.

## Discussion

In this study, we obtained novel plastome sequences of four species of *Renanthera* using next-generation sequencing techniques. Although there is a published report on the plastome sequence of *R. imschootiana* (Xin et al., 2020), information on its plastome sequence was not publicly available. Thus, it was not incorporated into this study. In this study, it was observed that the structure, gene content, and gene order in the four species of *Renanthera* were highly conserved. The plastome is generally maternal inherited in most angiosperms, including orchids, and usually comes with little recombination and a highly conserved structure across closely-related species (Wicke et al., 2011). Based on the study of Liu et al. (2020), the plastomes for members of Aeridinae were reported to range from 146,000 to 150,000 bp, except in two species, *Schoenorchis seidenfadenii* (142,859 bp) and *Uncifera acuminata* (145,191 bp). The species of *Renanthera* with the smallest plastome size, *R. imschootiana*, had a relatively smaller plastome size when compared to most of the members of Aeridinae; the three other species of *Renanthera* had a plastome size similar to most of the members of Aeridinae. The highly conserved number of genes present in the plastome of *Renanthera* suggested that the difference in plastome size could be due to the presence of indels in the intergenic spacer regions. However, similar to the rest of the members of Aeridinae, the overall GC content of *Renanthera* was not more than 37%.

The plastid *ndh* genes experienced independent loss in species of *Renanthera* (Table 2). Absence or pseudogenization of *ndh* genes in the plastomes of members of Aeridinae or Vandeae is considered a common phenomenon (Kim et al., 2020b; Jiang et al., 2022). The literature (Kim et al., 2015; Kim et al., 2020a) has reported that epiphytic orchids lose or pseudogenize their *ndh* genes when compared to terrestrial orchids, which is consistent with our findings for the four species of *Renanthera* in this study, which are epiphytes. Members of *Renanthera* rarely grows on the ground, they are usually epiphytic on tree trunks and/or lithophytic on rocks along valleys; thus, it is speculated that other species of *Renanthera* would undergo *ndh* gene loss, similar to those reported in this study. While it was proposed that the loss of *ndh* genes could be random in photosynthetic lineages, *ndh* gene loss is not only confined to Orchidaceae, but also reported in other autotrophic plants (Sabater, 2021). In general, *ndh* genes mediate cyclic electron transfer, which can protect plants from photoinhibition caused by various environmental stresses, and play an important role in maintaining efficient photosynthesis (Mi, 2016). The loss of *ndh* genes in the orchid plastome may be due to gene substitution or gene translocation from the plastome to the mitochondrial or nuclear genome (Lin et al., 2015). Usually, the loss of *ndh* genes in non-angiosperms, as in *Pinus* and *Welwitschia* (McCoy et al., 2008; Zeb et al., 2020), is not believed to be life-threatening. However, it could provide inverse effect to angiosperms as it will prevent plants from evolving and diversifying (Sabater, 2021), and will reduce the ecological adaptations of the species (Sun et al., 2020).

SSR are widely found in various species and are useful for studies of molecular evolution and genetic diversity (Doorduin et al., 2011). Of the 62–74 SSRs detected in the four species of *Renanthera*, the majority of them were identified from the LSC (73.2%) region, follow by SSC (19.4%) and IR (7.4%) region. Therefore, the distribution of SSRs in the chloroplast genome of *Renanthera* is uneven. The SSRs numbers in the LSC region of plastome of *R. coccinea* and *R. philippinensis* are 53 and 47, respectively. The distribution of SSR in the four species of *Renanthera* was consistent with that of most angiosperms (Xu et al., 2021; Sun et al., 2022; Xia et al., 2022).

The codon usage among the four plastomes of *Renanthera* was consistent across the four species; the CDSs of the four species of *Renanthera* had the same preferences in codon usage for all 20 amino acids, indicating that the nucleotide sequences in the CDS of the four species were identical, which is deemed accurate. This is because plastome sequences of closely related species have greater conservatism as they were inherited from their most recent common ancestor (Khan et al., 2021). Among these amino acids, leucine (*n* = 19,700) is the most frequently used, cysteine (*n* = 4,422) is the least universal amino acid in the cp genomes of these species. Alternatively, the high leucine frequency in the four plastomes can be attributed to the fact that leucine is largely needed in the chloroplasts due to its important function in photosynthesis-related metabolism (Knill et al., 2009). Cysteine, which was the least abundant amino acid among all the CDS analyzed, is a reactive element that can be considered toxic when it is accumulated above a certain level allowed by the host (Hildebrandt et al., 2015). This is consistent with the codon preference of other genera in the Orchidaceae, such as *Thuniopsis* and *Polystachya*. (Jiang et al., 2022; Li et al., 2022).

Contraction and expansion of the IR region is usually observed in most angiosperms (Li et al., 2019), including *Renanthera*. This was true when the 3’ end of the *rpl*22 gene of all four species of *Renanthera* that should be intact in the LSC region extended into the IRb region for 31–32 bp. The *ycf*1 gene in the SSC region of *R. citrina* was observed crossing over JSA, extending into the IRa region; while *ycf*1 of the remaining three species of *Renanthera* were intact in the SSC region. Generally, angiosperms retain the structure and size of the cp genome (Ebert and Peakall, 2010). However, due to evolutionary events such as contraction and expansion in the genome, variation can occur in the boundaries of inverted repeats (IRs) and single copy regions (LSC and SSC) (Raubeson et al., 2007) allowing certain genes to enter the IR or SC regions. Successive IR expansions have shown the importance of the JLA and JLB junctions for the analysis of evolutionary processes, providing clues about the origin and evolution of species (Zhang et al., 2013). Yet, in the case of *Renanthera*, although with the limited sampling size reported in this study, the finding suggested that the IR boundary shifts in *Renanthera* can be considered to be relatively minor, as this can be related to the small difference observed in the genome size across different species.

Sequence divergence analysis conducted on the four plastomes revealed that the IR region was highly conserved when compared to the single-copy regions. To our knowledge, there are no published reports on the molecular identification of the species of *Renanthera*. We speculated that this is either due to difficulty in species sampling, or the genus has not received attention. As molecular genetic tools could aid in the identification of the species of orchids and forensic detection at border controls (De and Pathak, 2018), the identification of useful gene regions in *Renanthera* will definitely contribute to safeguarding these orchids by security agencies. Molecular genetic tools such as DNA barcoding are among the promising techniques in the identification of species of land plants (Hollingsworth, 2011). The gene loci used in DNA barcoding usually contains sufficient informative sites that can delimit closely related species efficiently. The application of highly variable regions derived from the plastome sequence as potential DNA barcoding loci has been proven to be promising, as demonstrated in several findings (Vaughn et al., 2014; Lee et al., 2021). Three highly variable regions were identified in this study to facilitate marker development of *Renanthera*. It is noteworthy that an increased sampling size is needed to obtain reliable results (Li et al., 2015). The findings from this study may contribute to the development of DNA barcoding markers from *Renanthera*.

Despite being a valuable and threatened orchid, there are no reports on the phylogenetic relationships in *Renanthera*. According to the phylogenetic analysis using short gene sequences, *Renanthera* is monophyletic (Zou et al., 2015). However, due to small sampling size, we were not able to provide supporting evidence on this matter based on our phylogenetic analysis. Interestingly, the *Aerides* clade, where *Renanthera* is placed, was sister to Vanda clade when using the complete plastome sequence dataset. This finding was incongruent with the results by Zou et al. (2015) that used short gene sequences. Given that there are no known records on natural hybridization among species of Vandeae, the phylogenetic tree based on the plastome dataset is considered reliable (Liston et al., 2021). However, increased taxon samplings could reduce computational errors in phylogenetic tree reconstructions (Zwickl and Hillis, 2002); Zou et al. (2015) included 74 genera of Aeridinae in their analysis. In this study, only 24 genera of Aeridinae were included and the *Aerides* clade was represented by only one genus, *Renanthera*. Despite the fact that the phylogenetic analysis was well-resolved based on the plastome dataset, the finding by Zou et al. (2015) on the molecular placement of the *Aerides* clade in Aeridinae could be more reliable due to greater taxon sampling size. Thus, to ascertain the molecular placement of the *Aerides* clade in Aeridinae at the plastome level, it is suggested to include plastome data for several taxa of the *Aerides* clade such as species of *Aerides*, *Arachnis*, and *Rhynchostylis*.

## Conclusion

Our study revealed that the plastome of *Renanthera* is well-conserved across four species, *R. citrina, R. coccinea*, *R. imschootiana*, and *R. philippinensis*. Phylogenetic analysis based on the CDS sequences revealed that *Renanthera* is closely related to *Holcoglossum*, *Neofinetia*, *Pendulorchis*, and *Vanda* and the relationship of the four species of *Renanthera* was monophyletic. The genomic data obtained from this study will provide a useful resource for conservation, as well as to contributing in studies on the phylogeny and evolution of *Renanthera* and Orchidaceae.

## Data Availability

The data presented in the study are deposited in the GenBank of NCBI repository, accession numbers OK377033—OK377036..
